# Compare the Efficacy and Safety of Deferoxamine, Deferasirox, and Deferiprone in Patients With Sickle Cell Disease or Transfusion-Dependent Anemia: A Network Meta-Analysis of Randomized Control Trials

**DOI:** 10.7759/cureus.53644

**Published:** 2024-02-05

**Authors:** Divine Besong Arrey Agbor, Abhimanyu Karumanchi, Santoshini Adivi, Mohammed A Mohammed, Wajeeh Ur Rehman, Sandipkumar S Chaudhari, Thin M Soe, Neelum Ali

**Affiliations:** 1 Internal Medicine, Richmond University Medical Center, Staten Island, USA; 2 Medicine, Kamineni Institute of Medical Sciences, Hyderabad, IND; 3 Medicine, Non-Resident Indian (NRI) Medical College and Hospital, Guntur, IND; 4 General Practice, University of Florida, Gainesville, USA; 5 General Physician, Saidu Medical College, Khyber Medical University, Swat, PAK; 6 Cardiothoracic Surgery, University of Alabama at Birmingham, Birmingham, USA; 7 Family Medicine, University of North Dakota School of Medicine and Health Sciences, Fargo, USA; 8 Medicine, University of Medicine (1), Yangon, Yangon, MMR; 9 Internal Medicine, University of Health Sciences, Lahore, PAK

**Keywords:** systematic review and meta analysis, sickle cell disease, deferasirox, deferoxamine, deferiprone

## Abstract

This network meta-analysis was conducted with the aim of comparing the efficacy and safety of deferiprone (DFP), deferasirox (DFX), and deferoxamine (DFO) in individuals with sickle cell disease (SCD) or transfusion-dependent anemia. This systematic review and meta-analysis adhered to the “Preferred Reporting Items for Systematic Reviews and Meta-Analyses (PRISMA)” guidelines. The search was conducted on electronic databases, including PubMed, CINAHIL, and EMBASE, from the inception of databases to January 10, 2024. Outcomes assessed in this study included a change in liver iron concentration (LIC) and a change in ferritin from baseline. For safety analysis, adverse events were compared among three treatment groups. A total of five studies were included in this meta-analysis. The pooled analysis showed that the change in LIC and serum ferritin from baseline was not significantly different in patients with SCD or other anemias. In terms of adverse events, deferiprone was the safest among all. In conclusion, deferiprone demonstrated noninferiority to deferoxamine and deferasirox in measures of iron load, presenting a viable treatment option. Safety outcomes revealed deferasirox carried a higher risk of adverse events compared to deferiprone, supporting its favorable safety profile.

## Introduction and background

Sickle cell anaemia (SCA) stands as the most prevalent monogenic hereditary blood disorder, contributing to various life-threatening complications such as end-organ damage, kidney disease, heightened stroke risk, susceptibility to infections, and pulmonary issues [1-2]. Globally, an estimated 300,000 to 400,000 children are born with sickle cell disease (SCD) each year [3]. Many individuals with SCD necessitate occasional or ongoing red blood cell transfusions [4]. Despite its therapeutic value, blood transfusion introduces the risk of iron overload, posing a significant source of morbidity for these patients [5].

The body lacks a natural mechanism to eliminate excess iron, causing its rapid accumulation in the tissues of frequently transfused patients without iron chelation therapy [6]. Free iron proves toxic to cells, catalyzing the formation of free radicals and resulting in morbidity, including hepatic fibrosis, arrhythmias, congestive heart failure, various endocrinopathies, and, if left untreated, organ failure and death [7]. Consequently, when individuals require frequent blood transfusions, chelation treatment becomes imperative.

For the past four decades, deferoxamine (DFO) has stood as the preferred treatment for iron overload [8], with well-established efficacy in sickle cell disease patients [9]. However, limitations such as the need for overnight infusions and concerns about infection at the injection site have been identified, potentially leading to low compliance. Deferasirox (DFX) represents another approved option for managing iron overload in SCD. Administered orally as tablets or granules, DFX offers convenience but is associated with hepatic, gastrointestinal, and renal toxicities, raising concerns for SCD patients with preexisting renal impairment [10]. Deferasirox is an iron-chelating agent that binds to iron in a 2:1 ratio. It forms a stable complex with iron, and the resulting chelate is excreted primarily through the faeces [11].

Deferiprone (DFP) serves as an oral iron chelator, available in tablet and liquid forms, initially approved for treating iron overload in patients with thalassemia syndromes when other iron chelation therapies are inadequate [12]. Deferiprone is an orally active iron chelator that forms a 3:1 complex with iron. It binds to both ferric (Fe^3+^) and ferrous (Fe^2+^) ions, and the resulting complexes are excreted in the urine [13]. While its long-term efficacy and safety in that population are well documented [14], data in patients with SCD and other transfusion-dependent anaemias remain limited. Notably, there is a lack of head-to-head trials comparing DFO, DFP, and DFX in patients with SCD or transfusion-dependent anemia. This meta-analysis seeks to systematically compare the efficacy and safety profiles of deferiprone, deferasirox, and deferoxamine in individuals specifically diagnosed with SCD or those with transfusion-dependent anemia. The objective is to provide a comprehensive assessment of the therapeutic outcomes and safety considerations associated with each iron chelation therapy in the context of these specific hematologic conditions.

## Review

Methodology

Search Strategy

This systematic review and meta-analysis adhered to the “Preferred Reporting Items for Systematic Reviews and Meta-Analyses (PRISMA)” guidelines. The search was conducted on electronic databases including PubMed, CINAHIL, and EMBASE from the inception of databases to January 10, 2024, using the keywords "Deferiprone," "Deferoxamine," “Deferasirox,” “sickle cell disease,” and "anaemia," along with medical subject headings (MeSH) and relevant synonyms. We additionally searched Google Scholar to find additional studies relevant to the study objective. We also reviewed the studies included in previous review articles to find other eligible trials. Reference lists of all included studies were manually screened to identify additional studies relevant to the study topic. The language was restricted to English.

Selection Criteria

We included all randomized-control trials (RCTs) in this meta-analysis that compared any two of the three drugs (deferiprone, deferoxamine, and deferasirox) in patients with SCD and other anemia. We included studies that reported the following outcomes: change in liver iron concentration (LIC), change in ferritin from baseline, and safety events (number of adverse events, AE). We excluded studies that included patients other than those with SCD. We also excluded studies that did not report outcomes assessed in this present meta-analysis. We excluded observational studies, editorials, studies lacking comparison groups, and reviews. We stored all records gathered from online databases in EndNote X9. After removing duplicates, two authors independently reviewed titles and abstracts based on eligibility criteria. The full texts of the qualifying papers were also independently screened by the same two reviewers. Any disagreements at all stages were resolved by mutual consensus between the reviewers.

Data Extraction and Quality Assessment

A standardized extraction form was employed for gathering information from articles. The extracted data from the selected studies included details such as author names, publication year, sample size, characteristics of the study population, intervention protocols, and outcomes. The extracted data were cross-verified against the records to ensure accuracy.

To assess the risk of bias, two reviewers independently utilized the Cochrane Risk of Bias tool. This tool assesses studies across seven key domains: random sequence generation, allocation concealment, blinding of participants and personnel, blinding of outcome assessment, incomplete outcome data, selective reporting, and other biases. Each domain is rated as having a low, high, or unclear risk of bias. If all domains are assessed as low, the study is considered to have a low overall risk of bias. Conversely, if one or more domains have a high risk of bias, the study is deemed to have a high overall risk of bias. If there is insufficient information for a domain, the risk of bias for that domain is considered unclear. Any disagreements were resolved through discussion with a third investigator.

Data Analysis

The analysis of the results was conducted using Stata 17.0 software (StataCorp LLC, College Station, Texas, USA). For continuous variable outcome indicators, mean difference (MD) and 95% confidence interval (CI) were used as effect sizes, while odds ratio (OR) with a 95% CI was reported for categorical variables. Heterogeneity was evaluated through the Cochran Q test and I-square heterogeneity tests. Forest plots depicting outcome indicators were generated, and two-by-two comparisons were made to assess the efficacy and safety of each intervention.

To ensure the consistency of evidence for both direct and indirect comparisons, inconsistency tests were performed using nodal splitting. A p-value exceeding 0.05 indicated that the consistency assumption could be accepted at the overall level of each treatment. Surface under the cumulative ranking (SUCRA) scores were calculated and expressed as a percentage ranging from 0 (indicating the treatment as the worst) to 100% (indicating the treatment as the best). This score presented the likelihood of each intervention being considered the most effective. We were not able to assess publication bias as the total number of studies was less than 10.

Results

**Figure 1:**
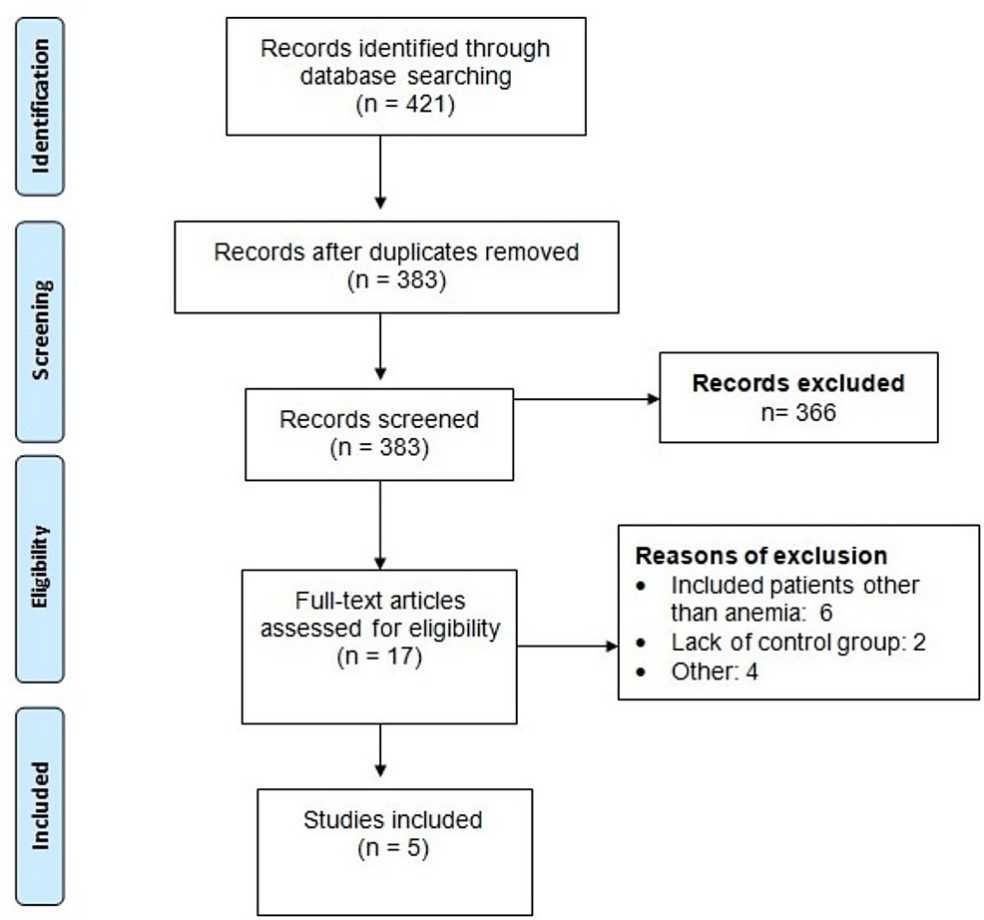
PRISMA flowchart of study selection

**Figure 2:**
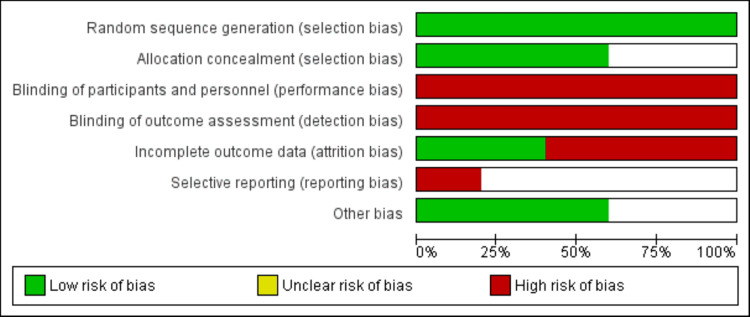
Quality assessment of included studies

Upon searching the databases, 421 articles were initially retrieved. After eliminating 38 duplicate studies, a further exclusion of 366 studies occurred based on the lack of relevance indicated by the title and abstract. Following a comprehensive assessment of full-text article eligibility, a total of five studies were deemed suitable for inclusion in the meta-analysis. The study selection process is illustrated in Figure 1, and Table 1 provides the characteristics of the included studies. Notably, all the studies were conducted at multiple centers, and a total of 1076 participants were encompassed across these studies. Figure 2 presents an assessment of the included studies. All studies were randomized, open-label, and non-blinded.

**Table 1:** Characteristics of included studies NR: not reported; n: number of studies; SCD: sickle cell disease

Study ID	Year	Origin	Condition	Groups	Sample size	Age (years)	Males (n)
Calvaruso et al. [9]	2014	Italy	SCD	Deferiprone	30	36.43	16
				Deferoxamine	30	35.83	14
Kwiatkowski et al. [15]	2022	8 Countries	SCD or a transfusion dependent anemia	Deferiprone	152	NR	83
				Deferoxamine	76	NR	38
Maggio et al. [16]	2020	6 Countries	SCD	Deferiprone	193	NR	113
				Deferasirox	197	NR	104
Vichinsky et al. [17]	2013	5 Countries	SCD	Deferoxamine	68	16.2	33
				Deferasirox	135	16.4	56
Vichinsky et al. [18]	2007	Canada and United States	SCD	Deferoxamine	63	16	28
				Deferasirox	132	15	52

Meta-Analysis of Efficacy Outcomes

**Figure 3:**
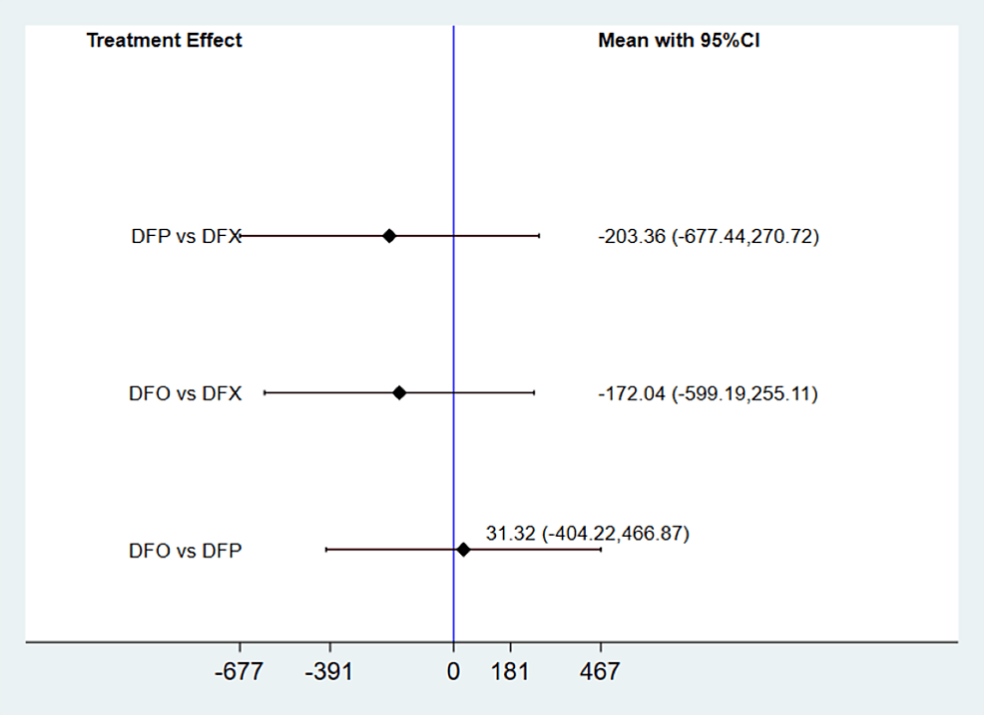
Comparison of three treatment groups on change in ferritin levels DFP: deferiprone; DFO: deferoxamine; DFX: deferasirox: CI: confidence interval

All studies, encompassing a total of 1076 participants, supplied appropriate data for assessing the changes in ferritin levels across the three treatment groups through a network meta-analysis. Comprehensive tests for both global and local inconsistencies revealed no significant inconsistencies, with p-values above 0.05. There was no significant heterogeneity (I-square: 39%, p-value: 0.13). As depicted in Figure 3, our findings indicate that the mean change in ferritin levels from baseline did not exhibit significant differences among deferiprone, deferoxamine, and deferasirox. The outcomes of the SUCRA analysis, presented in Table 2, indicate that deferiprone had the highest probability of being the most effective, followed by deferoxamine.

**Table 2:** SUCRA analysis of each outcome DFP: deferiprone; DFO: deferoxamine; DFX: deferasirox; SUCRA: surface under the cumulative ranking curve

Outcomes	DFP	DFX	DFO
LIC	76	54	52
Ferritin	78	26	54
Adverse events	98	20	0.1

**Figure 4:**
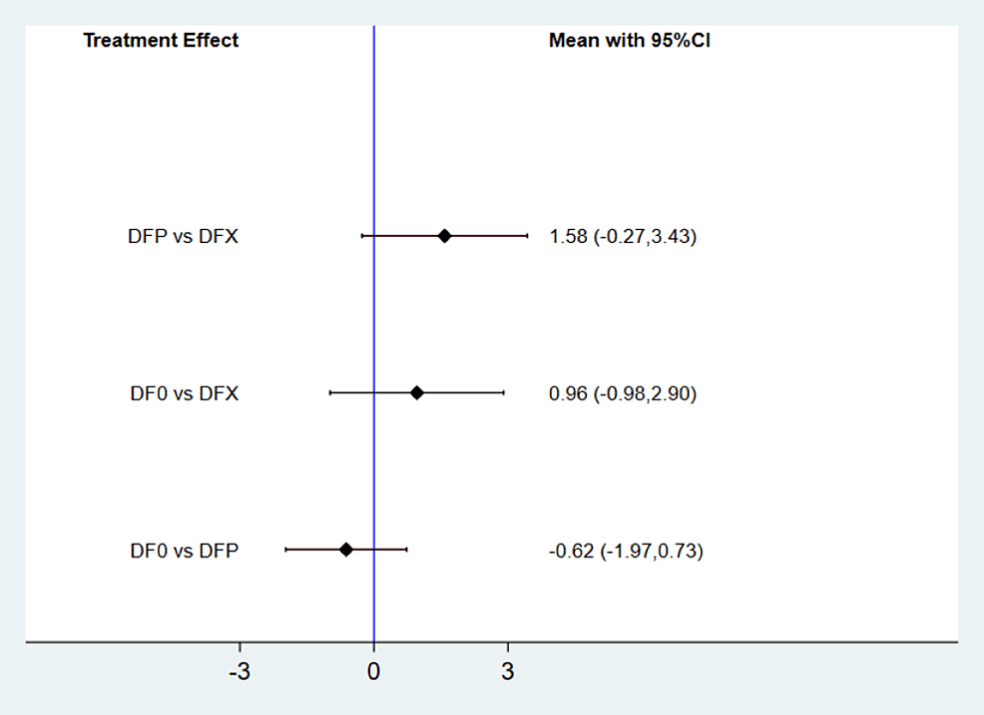
Comparison of change in liver iron concentration among three treatment groups DFP: deferiprone; DFO: deferoxamine; DFX: deferasirox: CI: confidence interval

The studies also presented data on the change in serum LIC from baseline, as illustrated in Figure 4. Global and local inconsistency tests showed no significant inconsistencies, with a p-value above 0.05. There was no significant heterogeneity (I-square: 16%, p-value: 0.27). The network meta-analysis indicated that there was no statistically significant difference in the change in LIC between deferiprone and deferoxamine, deferoxamine and deferasirox, and deferiprone and deferasirox. The SUCRA analysis results in Table 2 revealed that deferasirox had the highest probability of being the most effective, followed by deferoxamine and deferiprone, in terms of reducing LIC from baseline.

Meta-Analysis of Safety Outcomes

**Figure 5:**
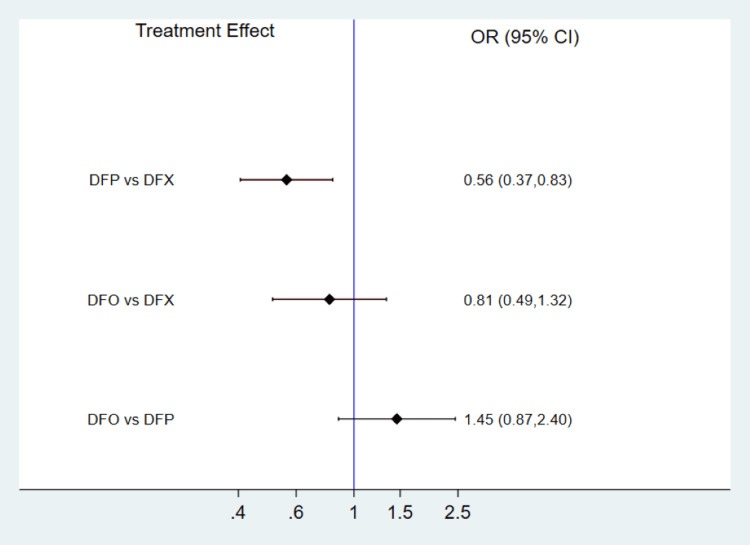
Comparison of adverse events among three treatment groups DFP: deferiprone; DFO: deferoxamine; DFX: deferasirox; OR: odds ratio; CI: confidence interval

We compared the risk of adverse events among the three treatment groups via a network meta-analysis. No significant inconsistency was detected by the global and local inconsistency tests, as the p-value was more than 0.05. Therefore, the consistency model was used to calculate the pooled estimate. There was no significant heterogeneity (I-square: 20%, p-value: 0.31). A network meta-analysis suggested that the risk of adverse events was significantly higher in patients receiving deferasirox compared to deferiprone, as shown in Figure 5. However, the risk of adverse events was not significantly different between deferiprone and deferoxamine and deferoxamine and deferasirox. Results of the SUCRA analysis revealed that deferiprone had the highest probability of being safest, followed by deferoxamine and deferasirox.

Discussion

In this meta-analysis, deferiprone demonstrated comparable efficacy to deferoxamine and deferasirox in both measures of iron load change in LIC and serum ferritin from baseline in patients with SCD or other anemias. Deferiprone exhibited good tolerability, with an overall safety profile deemed acceptable and largely in line with observations in patients with thalassemia syndromes [12,19]. The findings of this meta-analysis highlight that, in terms of safety, deferiprone stands out as the preferred option compared to both deferoxamine and deferasirox.

SCA patients undergoing regular transfusions face the risk of iron overload associated with transfusions. Deferoxamine has been used as a treatment option in the last several years [8]. Nevertheless, concerns regarding compliance and adverse effects linked to deferoxamine have sparked interest in exploring alternative therapies. Deferasirox, administered orally, presents a potential avenue for enhancing patient outcomes by promoting better adherence [20]. However, it is noteworthy that deferasirox is associated with a higher incidence of adverse events compared to deferiprone in this study.

The study conducted by Maggio et al. reported that non-serious events were significantly higher in patients receiving deferasirox compared to patients receiving deferiprone [16]. The study also reported that renal function abnormalities were common in the deferasirox group. Therefore, this drug needs to be used cautiously in patients with renal and hepatic abnormalities. However, the Food and Drug Administration Authority (FDA) conducted the RCT that compared the efficacy and safety of deferiprone with deferoxamine [16].

One of the adverse events associated with deferiprone is neutropenia, as reported by two of the included studies in this review. In the study conducted by Kwiatkowski et al., milder neutropenia was reported in 2.6% of patients who received deferiprone. Most neutropenia cases are resolved within 4 to 12 days [15]. However, Maggio et al. reported that the rate of mild or moderate neutropenia was higher in the deferiprone group (9%) compared to the deferasirox group (6%), but the difference was statistically insignificant [16]. Considering the association of neutropenia with deferiprone therapy, the initial FDA marketing authorization application for deferiprone recommended weekly monitoring of the absolute neutrophil count (ANC). This monitoring practice has been outlined in the product information since 2011 [21].

Adherence to the prescribed treatment plan plays a crucial role in ensuring treatment effectiveness and achieving a long-term reduction in body iron levels. Previous studies have indicated a higher level of compliance with deferiprone compared to deferoxamine [22]. In our study, 68.9% of patients in the deferiprone group and 78.9% in the deferoxamine group met the criteria for treatment compliance, although this difference was not statistically significant. Initially, deferiprone was exclusively available and approved for a three-times-daily dosing schedule. However, real-world data and input from physicians and patients suggest that the three-times-daily deferiprone regimen may be inconvenient for some individuals, particularly school-age children who often miss the midday dose. A twice-daily treatment schedule is now approved in the United States [23], and this change is anticipated to enhance overall compliance [24].

The oral solution form of deferiprone is an alternative, and it has been linked to fewer gastrointestinal AEs when compared to the tablet form [25-26]. This aspect may enhance compliance, particularly for patients encountering adverse gastrointestinal symptoms with the tablets. Additionally, it can be prescribed to individuals who have experienced intolerance to deferasirox due to gastrointestinal adverse events related to that medication. Furthermore, educating patients about treatment side effects could enhance their understanding and potentially contribute to improved treatment compliance.

This meta-analysis has some inherent limitations. First, the inclusion of only five RCTs in this review may diminish the overall pooling effect. Additionally, the meta-analysis concentrated on efficacy measures, specifically iron overload, necessitating a separate evaluation of the long-term safety associated with these iron-chelating agents. Moreover, the three studies exhibited heterogeneity in their diverse dosing regimens and assessment period lengths. Third, the absence of individual-level data, such as age and comorbidities, hindered the ability to conduct subgroup analysis.

Patients with SCA and other hemoglobinopathies often experience the complications of chronic iron overload. There is a need for additional large-scale, multicenter, investigator-sponsored studies specifically comparing deferasirox with deferiprone and deferoxamine to enhance our understanding of the efficacy and safety of deferasirox in individuals with SCA. Furthermore, research exploring various dosing schedules and considering sickle cell disease comprehensively is warranted.

## Conclusions

In conclusion, our meta-analysis, encompassing 1076 participants from five selected studies, evaluated the efficacy and safety of iron-chelating agents in individuals with SCD or other anemias. Deferiprone demonstrated noninferiority to deferoxamine and deferasirox in measures of iron load, presenting a viable treatment option. Safety outcomes revealed deferasirox carried a higher risk of adverse events compared to deferiprone, supporting its favourable safety profile. Future large, multicenter studies comparing deferasirox, deferiprone, and deferoxamine, with a focus on diverse dosing schedules and populations with sickle cell disease, are essential for comprehensive understanding and further advancement in iron overload management."
